# Trends in hospital admissions among children with asthma in Spain (2011–2020)

**DOI:** 10.1007/s00431-023-04873-w

**Published:** 2023-03-14

**Authors:** Natalia Gutierrez-Albaladejo, Rodrigo Jimenez-Garcia, Romana Albaladejo-Vicente, Rosa Villanueva-Orbaiz, Javier de-Miguel-Diez, Concepción Noriega, Ana Lopez-de-Andres

**Affiliations:** 1 Allergy Department, Hospital Universitario Fundación de Alcorcón, 28922 Alcorcón Madrid, Spain; 2 Department of Public Health & Maternal and Child Health, Faculty of Medicine, Universidad Complutense de Madrid, 28040 Madrid, Spain; 3 Respiratory Department, Hospital General Universitario Gregorio Marañón, Instituto de Investigación Sanitaria Gregorio Marañón (IiSGM), Faculty of Medicine, Universidad Complutense de Madrid, 28040 Madrid, Spain; 4 Department of Nursery and Physiotherapy, Faculty of Medicine and Health Sciences, University of Alcalá, 28871 Alcalá de Henares, Madrid, Spain

**Keywords:** Asthma, Exacerbations, Incidence, Hospitalization, Pediatric, Mortality, COVID-19

## Abstract

The purpose of this study is to describe and assess changes in incidence, clinical conditions, use of mechanical ventilation, length of hospital stay (LOHS), and in-hospital mortality (IHM) among children hospitalized with asthma in Spain from 2011 to 2020. We analyzed children aged 0 to 15 years hospitalized with an ICD code for asthma included in the Spanish National Hospital Discharge Database (SNHDD). The analysis was conducted for asthma as the primary diagnosis and with asthma in any diagnosis position. Joinpoint regression was used to assess time trends in incidence. We included a total of 85,664 children hospitalized with asthma; of these, 46,727 (54.55%) had asthma coded as the primary diagnosis. The number of boys was higher than the number of girls, irrespective of age group or diagnostic position. The frequency of asthma as primary diagnosis decreased from 55.7% in 2011 to 43.96% in 2020 (*p* < 0.001). The incidence of hospitalizations because of asthma decreased significantly from 2011 to 2020, with a faster decrease from 2018 onwards. Over time, the proportion of older children increased. In the year 2020, only 55 children had codes for asthma and COVID-19 in their discharge report, and this infection had no effect on hospitalizations this year. A significant increase in the use of non-invasive ventilation (NIV) was observed over time. Irrespective of the diagnostic position, LOHS and IHM remained stable over time, with the IHM under 0.1%.

*  Conclusion*: Our results show a decrease in the incidence of hospital admissions with asthma either as the primary diagnosis or in any position. The age of children hospitalized seems to be increasing as the use of NIV. Better management of the disease from primary care and the emergency department as is the use of NIV could explain the reduction in incidence.

**Table:** 

**What is Known:**
• *Asthma is the most common chronic respiratory in childhood in high income countries.*
• *The incidence of hospital admissions with asthma and associated factors is one of the best sources of information on morbidity trends and prognosis.*
**What is New:**
• *The incidence of hospital admissions for asthma in Spain decreased in children between 2011 and 2020 with a more frequent use of non-invasive mechanical ventilation and low mortality rates.*
• *COVID-19 did not cause an increase in admissions with asthma in the year 2020.*

**Supplementary Information:**

The online version contains supplementary material available at 10.1007/s00431-023-04873-w.

## Introduction

Asthma is the most common chronic respiratory disease in the world, and in 2019, it was estimated to affect 262 million people. There are wide geographical variations in the prevalence of asthma, with higher rates in higher-income countries where it is the most common chronic disease in childhood [1–6]. However, a significant decrease in the prevalence of asthma has been observed in these countries over the last decade [3–5]. Asthma prevalence rates in children under 18 years increased in the USA between 2001 and 2009 (from 8.7 to 9.7%) and then declined, with a prevalence of 7.5% in 2018 [4, 5]. In Spain, asthma symptoms have become highly prevalent, increasing in adolescents (15.3% at 13–14 years) and stabilizing in schoolchildren (10.4% at 6–7 years) [7]. Regarding sex differences, asthma is more prevalent in boys (9.2%) than in girls before the onset of puberty (7.4%), with this trend reversing in adolescence and thereafter [8, 9].

The incidence of hospital admissions with asthma and associated factors is one of the best sources of information on morbidity trends and prognosis [10].

Some Spanish authors have suggested that an increase in the use of non-invasive ventilation (NIV) at the emergency room in pediatric and adolescent asthma patients could be related to a decrease in the incidence of hospital admissions due to asthma exacerbations in this population [11, 12].

Recent works that have assessed the possible association between asthma and COVID-19 concluded that asthmatic patients do not seem to have an increased risk of admission for COVID-19 [13].

When asthma treatment is inadequate, the complications associated with the disease can result in more frequent exacerbations and hospitalizations, which can in turn lead to increased hospital stay and mortality. However, deaths from asthma in children are rare (range, 0.0–0.7 cases per 100 000) and are associated with severe asthma symptoms and hospital admissions [14, 15].

The aims of this study were to analyze trends in the admissions of children to hospital with asthma in Spain between 2011 and 2020 and to identify possible changes in incidence, demographic characteristics, clinical conditions, and hospital outcomes such as length of hospital stay (LOHS) and in-hospital mortality (IHM). The analysis was conducted with asthma as the primary diagnosis and with asthma in any diagnostic position.

## Methods and subjects

We conducted an observational retrospective epidemiological study using the Spanish National Hospital Discharge Database (SNHDD), which is managed by the Spanish Ministry of Health. Since all private and public hospitals provide data, over 95% of hospitalizations are included. Clinical conditions and diagnostic and therapeutic procedures performed during the admission are coded using the International Classification of Diseases (ICD). The Ninth Revision (ICD-9) was used from implementation in 1992 up to the year 2015. In the year 2016, ICD-9 was replaced by the Tenth Revision (ICD-10) [16, 17].

### Study population and variables

We selected all admissions of children aged 0 to 15 years with a diagnostic code for asthma in any position (see Supplementary Table 1 for ICD-9 and ICD-10 codes).

The study population was analyzed using asthma as the primary diagnosis and with asthma in any diagnostic position. We analyzed asthma in “any diagnostic position” to assess possible changes in the way the disease was coded in the SNHDD over time and to obtain an overview of the importance of asthma in children.

According to the SNHDD methodology, the primary diagnosis is the clinical condition that is considered, by the physician responsible for the discharge report to have been the main reason for the child being admitted to hospital [16, 17]. In previous investigations, when “Asthma” was coded in this position, the admission was defined as an “Asthma exacerbation” [2, 11].

For the purposes of this study, we excluded children with missing data on age, sex, LOHS, and discharge destination, with no imputation of missing data.

Our main study variables were the incidence of hospitalizations for asthma and the IHM. The incidence for each year from 2011 to 2020 was estimated by sex and three age groups (0–5 years; 6 to 10 years; and 11 to 15 years). Yearly populations for incidence calculations were obtained from the Spanish National Statistics Institute [18].

Study covariates included the presence of pneumonia, influenza, and COVID-19 (only for the year 2020). We also described the use of invasive mechanical ventilation (IMV) and NIV. The ICD codes for these conditions and procedures are defined in Supplementary Table 1.

### Statistical analysis

The incidence rates were expressed per 100,000 children. Joinpoint log-linear regression was applied to identify the years in which changes in tendency occurred in the rates for hospital admissions for asthma as either the primary diagnosis or in any diagnostic position, according to age group and sex. Joinpoint regression provides the annual percentage change (APC) in each of the periods delimited by the points of change, and tests whether an apparent change in trend is statistically significant [19].

Study variables were described using means with standard deviations and total numbers with relative frequencies (expressed as percentages) for continuous and categorical variables, respectively. To assess the time trend from 2011 to 2020 for categorical variables, we used the Cochran-Mantel–Haenszel test (age group) or Cochran-Armitage test (binary variables). In the case of LOHS, we used the Jonckheere-Terpstra test.

Finally, we constructed a multivariable logistic regression model to identify which study variables were independently associated with IHM when asthma was coded in any diagnostic position. The models were constructed using the methods described by Hosmer et al. [20].

Data were analyzed using Joinpoint Regression Program, version 4.0.4 [21], and Stata version 14 (Stata, College Station, Texas, USA). Statistical significance was set at *p* < 0.05 (two-sided).

### Ethical aspects

The Spanish Ministry of Health evaluated the protocol of our investigation prior to providing us with the SNHDD database. All data received were anonymized. For the previous reasons and according to Spanish legislation, the requirement for ethical approval was not necessary. Any investigator can request the SNHDD from the Spanish Ministry of Health by completing an application form [22].

## Results

Between 2011 and 2020, 86,416 children were hospitalized with a code for asthma in any diagnostic position. Of those, 752 (0.87%) were excluded for the following reasons: missing data for age (*n* = 21; < 0.1%), sex (*n* = 15; < 0.1%), LOHS (*n* = 562; 0.65%), and discharge destination (*n* = 154; 0.18%). Therefore, the final study population included 85,664 children. Over the 10-year period, 46,727 (54.55%) children had asthma coded as their primary diagnosis. The proportion of children with a primary diagnosis of asthma decreased significantly from 55.70% in 2011 to 43.96% in 2019–2020 (*p* < 0.001) (Table 1).

**Table 1:** Children hospitalized with asthma as primary diagnosis or in any diagnosis position in Spain from 2011 to 2020 according to age groups and sex

	Sex	Age group	**2011**	**2012**	**2013**	**2014**	**2015**	**2016**	**2017**	**2018**	**2019**	**2020**
Primary diagnosis	Boys^a^	0–5 years, *n* (%)	2319(69.12)	2304(67.29)	2192(64.17)	1999(63.36)	2077(63.38)	1756(60.22)	1444(56.49)	1564(58.84)	1183(55.25)	667(53.19)
		6–10 years, *n* (%)	758(22.59)	836(24.42)	919(26.9)	837(26.53)	880(26.85)	832(28.53)	781(30.56)	798(30.02)	663(30.97)	419(33.41)
		11–15 years, *n* (%)	278(8.29)	284(8.29)	305(8.93)	319(10.11)	320(9.77)	328(11.25)	331(12.95)	296(11.14)	295(13.78)	168(13.4)
		0–15 years, *n* (%)	3355(100)	3424(100)	3416(100)	3155(100)	3277(100)	2916(100)	2556(100)	2658(100)	2141(100)	1254(100)
	Girls^a^	0–5 years, *n* (%)	1434(66.7)	1353(65.43)	1267(60.22)	1295(61.61)	1189(56.57)	1127(55.03)	948(52.03)	994(54.23)	778(52.75)	447(51.32)
		6–10 years, *n* (%)	529(24.6)	496(23.98)	608(28.9)	550(26.17)	577(27.45)	615(30.03)	581(31.89)	585(31.91)	442(29.97)	288(33.07)
		11–15 years, *n* (%)	187(8.7)	219(10.59)	229(10.88)	257(12.23)	336(15.98)	306(14.94)	293(16.08)	254(13.86)	255(17.29)	136(15.61)
		0–15 years, *n* (%)	2150(100)	2068(100)	2104(100)	2102(100)	2102(100)	2048(100)	1822(100)	1833(100)	1475(100)	871(100)
	Both sexes^a^	0–5 years, *n* (%)	3753(68.17)	3657(66.59)	3459(62.66)	3294(62.66)	3266(60.72)	2883(58.08)	2392(54.64)	2558(56.96)	1961(54.23)	1114(52.42)
		6–10 years, *n* (%)	1287(23.38)	1332(24.25)	1527(27.66)	1387(26.38)	1457(27.09)	1447(29.15)	1362(31.11)	1383(30.79)	1105(30.56)	707(33.27)
		11–15 years, *n* (%)	465(8.45)	503(9.16)	534(9.67)	576(10.96)	656(12.2)	634(12.77)	624(14.25)	550(12.25)	550(15.21)	304(14.31)
		0–15 years, *n* (%)	5505(100)	5492(100)	5520(100)	5257(100)	5379(100)	4964(100)	4378(100)	4491(100)	3616(100)	2125(100)
Any diagnosis position	Boys^a^	0–5 years, *n* (%)	3420(56.42)	3247(54.39)	3119(51.06)	2968(50.34)	2948(49.01)	2467(49.61)	2098(44.23)	2225(46.96)	1813(42.87)	1053(37.04)
		6–10 years, *n* (%)	1534(25.31)	1548(25.93)	1768(28.94)	1700(28.83)	1794(29.83)	1457(29.3)	1453(30.63)	1425(30.08)	1258(29.75)	899(31.62)
		11–15 years, *n* (%)	1108(18.28)	1175(19.68)	1222(20)	1228(20.83)	1273(21.16)	1049(21.09)	1192(25.13)	1088(22.96)	1158(27.38)	891(31.34)
		0–15 years, *n* (%)	6062(100)	5970(100)	6109(100)	5896(100)	6015(100)	4973(100)	4743(100)	4738(100)	4229(100)	2843(100)
	Girls^a^	0–5 years, *n* (%)	2170(56.78)	1923(52.95)	1867(48.81)	1909(48.97)	1731(45.44)	1566(45.44)	1366(41.39)	1462(43.03)	1243(41.92)	702(35.26)
		6–10 years, *n* (%)	978(25.59)	966(26.6)	1114(29.12)	1061(27.22)	1076(28.25)	1038(30.12)	1009(30.58)	1046(30.78)	845(28.5)	604(30.34)
		11–15 years, *n* (%)	674(17.63)	743(20.46)	844(22.07)	928(23.81)	1002(26.31)	842(24.43)	925(28.03)	890(26.19)	877(29.58)	685(34.4)
		0–15 years, *n* (%)	3822(100)	3632(100)	3825(100)	3898(100)	3809(100)	3446(100)	3300(100)	3398(100)	2965(100)	1991(100)
	Both sexes^a^	0–5 years, *n* (%)	5590(56.56)	5170(53.84)	4986(50.19)	4877(49.8)	4679(47.63)	4033(47.9)	3464(43.07)	3687(45.32)	3056(42.48)	1755(36.31)
		6–10 years, *n* (%)	2512(25.41)	2514(26.18)	2882(29.01)	2761(28.19)	2870(29.21)	2495(29.64)	2462(30.61)	2471(30.37)	2103(29.23)	1503(31.09)
		11–15 years, *n* (%)	1782(18.03)	1918(19.98)	2066(20.8)	2156(22.01)	2275(23.16)	1891(22.46)	2117(26.32)	1978(24.31)	2035(28.29)	1576(32.6)
		0–15 years, *n* (%)	9884(100)	9602(100)	9934(100)	9794(100)	9824(100)	8419(100)	8043(100)	8136(100)	7194(100)	4834(100)
Primary diagnosis		%	55.7	57.2	55.57	53.68	54.75	58.96	54.43	55.2	50.26	43.96

^a^Significant change (*p* < 0.001) in the distribution by age groups from 2011 to 2020 (Cochran-Mantel–Haenszel statistic)

### Time trends in the incidence and characteristics of children hospitalized with asthma as the primary diagnosis

Table 1 shows the total number of hospitalizations for children with asthma as the primary diagnosis and in any diagnostic position according to age group and sex. The number of children with a primary diagnosis of asthma decreased sharply from 2011 to 2020 for all age groups and both sexes. The age group with the highest proportion of hospitalizations (both boys and girls) was the 0- to 5-year group. However, a significant increase in the older age groups was observed over time. From 2011 to 2020, the percentage of children with a diagnosis of asthma aged 0–5 years decreased from 68.17 to 52.42%, whereas that of groups aged 6–10 years and 11–15 years increased from 23.38 to 33.27% and from 8.45 to 14.31%, respectively (*p* < 0.001).

Over the entire period, boys outnumbered girls (28,152 [60.24%] vs. 18,575 [39.76%]). In all the years analyzed and for all age groups, the number of boys was higher than the number of girls.

**Fig. 1:**
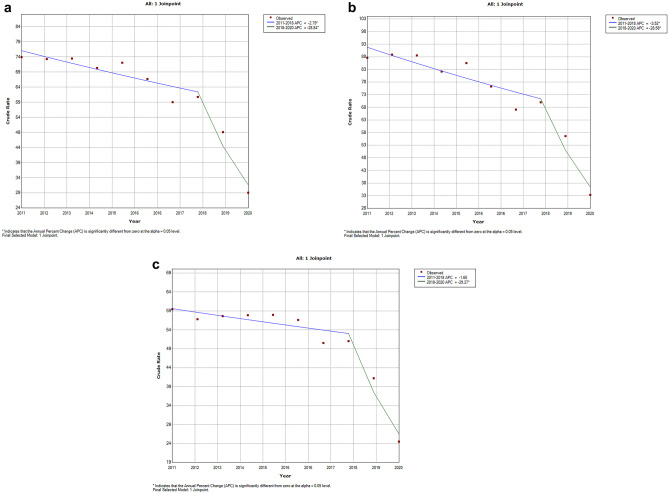
Time trend from 2011 to 2020 in incidence of hospitalizations for asthma as the primary diagnosis according to the joinpoint analysis for both sexes (**a**), boys (**b**), and girls (**c**)

Figure 1 shows the changes in incidence of hospitalizations for asthma as the primary diagnosis according to the joinpoint analysis for both sexes (Fig. 1a), boys (Fig. 1b), and girls (Fig. 1c).

As can be seen in Fig. 1a, the incidence of hospitalizations declined significantly in children, with an APC of 2.79% from 2011 to 2018 and of 28.84% from 2018 until 2020. The trend for boys and girls was almost identical (Fig. 1b, c), with a constant reduction over time that became more intense from 2018 onwards.

Table 2 shows the prevalence of pneumonia, influenza, COVID-19, use of mechanical ventilation, LOHS, and IHM among children hospitalized with asthma as the primary diagnosis in Spain from 2011 to 2020 according to sex. The frequency of concomitant pneumonia was low over all the years studied, with figures of around 2–4% for both sexes and no significant changes over time. Influenza was diagnosed even less frequently, with figures below 1% in boys and girls and in every year analyzed. In the year 2020 in Spain, only 7 children (3 boys and 4 girls) were hospitalized with a primary diagnosis of asthma and COVID-19.

**Table 2:** Prevalence of pneumonia, influenza, COVID-19, use of mechanical ventilation, length of hospital stays, and in-hospital mortality among children hospitalized with asthma as primary diagnosis in Spain from 2011 to 2020 according to sex

		**2011**	**2012**	**2013**	**2014**	**2015**	**2016**	**2017**	**2018**	**2019**	**2020**	***p***
Boys	Pneumonia, *n* (%)	86(2.56)	82(2.39)	100(2.93)	72(2.28)	83(2.53)	96(3.29)	72(2.82)	81(3.05)	89(4.16)	34(2.71)	0.064
	Influenza, *n* (%)	12(0.36)	12(0.35)	9(0.26)	14(0.44)	9(0.27)	12(0.41)	7(0.27)	16(0.6)	17(0.79)	5(0.4)	0.179
	COVID-19, *n* (%)	NA	NA	NA	NA	NA	NA	NA	NA	NA	3(0.24)	NA
	IMV, *n* (%)	17(0.51)	18(0.53)	14(0.41)	8(0.25)	16(0.49)	15(0.51)	7(0.27)	7(0.26)	9(0.42)	5(0.4)	0.577
	NIMV, *n* (%)	52(1.55)	83(2.42)	82(2.4)	83(2.63)	97(2.96)	79(2.71)	46(1.8)	65(2.45)	59(2.76)	41(3.27)	0.003
	LOHS, mean (SD)	3.12(2.35)	3.16(3.29)	3.02(1.97)	3.04(1.96)	3(2)	3.25(2.06)	3.21(2.12)	3.07(2)	3.23(2.14)	2.99(2.31)	0.07
	IHM, *n* (%)	0(0)	1(0.03)	1(0.03)	0(0)	2(0.06)	1(0.03)	0(0)	0(0)	1(0.05)	0(0)	0.712
Girls	Pneumonia, *n* (%)	65(3.02)	68(3.29)	80(3.8)	80(3.81)	64(3.04)	102(4.98)	75(4.12)	73(3.98)	65(4.41)	35(4.02)	0.091
	Influenza, *n* (%)	7(0.33)	5(0.24)	5(0.24)	8(0.38)	9(0.43)	17(0.83)	10(0.55)	8(0.44)	9(0.61)	7(0.8)	0.288
	COVID-19, *n* (%)	NA	NA	NA	NA	NA	NA	NA	NA	NA	4(0.46)	NA
	IMV, *n* (%)	8(0.37)	8(0.39)	13(0.62)	9(0.43)	6(0.29)	11(0.54)	6(0.33)	4(0.22)	1(0.07)	5(0.57)	0.269
	NIMV, *n* (%)	34(1.58)	57(2.76)	45(2.14)	65(3.09)	64(3.04)	50(2.44)	36(1.98)	37(2.02)	37(2.51)	32(3.67)	0.005
	LOHS, mean (SD)	3.18(2.24)	3.26(2.68)	3.28(2.2)	3.31(2.24)	3.2(2.58)	3.47(2.78)	3.4(2.34)	3.28(2.18)	3.37(2.34)	3.23(2.37)	0.092
	IHM, *n* (%)	0(0)	0(0)	2(0.1)	0(0)	1(0.05)	0(0)	0(0)	0(0)	0(0)	0(0)	0.23
Both sexes	Pneumonia, *n* (%)	151(2.74)	150(2.73)	180(3.26)	152(2.89)	147(2.73)	198(3.99)	147(3.36)	154(3.43)	154(4.26)	69(3.25)	0
	Influenza, *n* (%)	19(0.35)	17(0.31)	14(0.25)	22(0.42)	18(0.33)	29(0.58)	17(0.39)	24(0.53)	26(0.72)	12(0.56)	0.016
	COVID-19, *n* (%)	NA	NA	NA	NA	NA	NA	NA	NA	NA	7(0.33)	NA
	IMV, *n* (%)	25(0.45)	26(0.47)	27(0.49)	17(0.32)	22(0.41)	26(0.52)	13(0.3)	11(0.24)	10(0.28)	10(0.47)	0.314
	NIMV, *n* (%)	86(1.56)	140(2.55)	127(2.3)	148(2.82)	161(2.99)	129(2.6)	82(1.87)	102(2.27)	96(2.65)	73(3.44)	< 0.001
	LOHS, mean (SD)	3.14(2.31)	3.2(3.08)	3.12(2.06)	3.15(2.08)	3.08(2.24)	3.34(2.39)	3.29(2.22)	3.16(2.07)	3.28(2.23)	3.09(2.34)	0.051
	IHM, *n* (%)	0(0)	1(0.02)	3(0.05)	0(0)	3(0.06)	1(0.02)	0(0)	0(0)	1(0.03)	0(0)	0.238

*p* value for the trend from 2011 to 2020 (Cochran-Armitage tests for trend for categorical variables and Jonckheere-Terpstra test for LOHS)

*NA* not applicable, *IMV* invasive mechanical ventilation, *NIMV* non-invasive mechanical ventilation, *LOHS* length of hospital stay, *IHM* in-hospital mortality

We detected a significant increase in the use of NIV in both sexes (1.55% in boys and 1.56% in girls in 2011 vs. 3.27% and 3.67% in 2020). IMV was very infrequent (< 0.7%), with no variation over time.

In our study, the mean LOHS remained stable at around 3 days from 2011 to 2020. IHM was very low, with no significant changes detected over the study period. Nine children died in hospital (6 boys and 3 girls).

### Time trends in the incidence and characteristics of children hospitalized with asthma in any diagnostic position

A total of 85,664 children were hospitalized with asthma in any diagnostic position in Spain from 2011 to 2020. The highest figure corresponds to the year 2013, with 9934 children, and the lowest to the year 2020, with only 4834 children (Table 1). As mentioned above, the number of hospitalizations for asthma in the primary diagnostic position decreased for all age groups and both sexes. The proportion of children (boys and girls) in the 0- to 5-year group decreased over time as the older age groups became proportionally larger. Boys accounted for 60.21% (51,578) of the study population and girls for 39.79% (34,086).

**Fig. 2:**
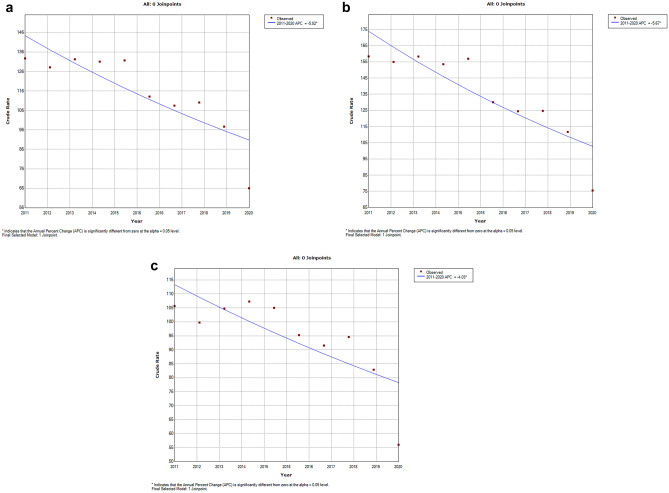
Time trend from 2011 to 2020 in incidence of hospitalizations for asthma in any diagnostic position according to the joinpoint analysis for both sexes (**a**), boys (**b**), and girls (**c**)

Trends in incidence analyzed using joinpoint regression are shown in Fig. 2. For all the children (Fig. 2a), incidence decreased over time, with an APC of 5.02% from the year 2011 to 2020. Trends also decreased constantly for both boys and girls from 2011 to 2020, with a similar pattern (Fig. 2b, c).

As can be seen in Table 3, the diagnosis of pneumonia appeared much more frequently when asthma was present in any diagnostic position than when only the primary position was analyzed. The prevalence of pneumonia among girls ranged from 7 to 11%, with a significant decrease over time. Lower figures were reported for boys, ranging from 6 to 9%, and a reduction was also observed between 2011 and 2020 (*p* < 0.001).

**Table 3:** Prevalence of pneumonia, influenza, COVID-19, use of mechanical ventilation, length of hospital stays, and in-hospital mortality among children hospitalized with asthma in any diagnosis position in Spain from 2011 to 2020 according to sex

		**2011**	**2012**	**2013**	**2014**	**2015**	**2016**	**2017**	**2018**	**2019**	**2020**	***p***
Boys	Pneumonia, *n* (%)	512 (8.45)	384(6.43)	391(6.4)	431(7.31)	411(6.83)	332(6.68)	303(6.39)	313(6.61)	331(7.83)	162(5.7)	< 0.001
	Influenza, *n* (%)	48(0.79)	33(0.55)	28(0.46)	47(0.8)	40(0.67)	68(1.37)	19(0.4)	57(1.2)	70(1.66)	54(1.9)	< 0.001
	COVID-19, *n* (%)	NA	NA	NA	NA	NA	NA	NA	NA	NA	32(1.13)	< 0.001
	IMV, *n* (%)	45(0.74)	37(0.62)	33(0.54)	27(0.46)	40(0.67)	27(0.54)	24(0.51)	21(0.44)	29(0.69)	18(0.63)	0.496
	NIMV, *n* (%)	79(1.3)	115(1.93)	126(2.06)	132(2.24)	151(2.51)	104(2.09)	70(1.48)	83(1.75)	86(2.03)	63(2.22)	< 0.001
	LOHS, mean (SD)	3.48(3.77)	3.44(3.98)	3.34(3.42)	3.46(4.18)	3.4(3.82)	3.48(3.42)	3.54(3.45)	3.44(3.68)	3.57(5.27)	3.35(3.36)	0.068
	IHM, *n* (%)	3(0.05)	3(0.05)	4(0.07)	2(0.03)	8(0.13)	3(0.06)	2(0.04)	2(0.04)	1(0.02)	2(0.07)	0.539
Girls	Pneumonia, *n* (%)	421(11.02)	331(9.11)	373(9.75)	380(9.75)	352(9.24)	329(9.55)	305(9.24)	300(8.83)	300(10.12)	140(7.03)	0.001
	Influenza, *n* (%)	27(0.71)	21(0.58)	14(0.37)	24(0.62)	23(0.6)	46(1.33)	21(0.64)	38(1.12)	34(1.15)	57(2.86)	< 0.001
	COVID-19, *n* (%)	NA	NA	NA	NA	NA	NA	NA	NA	NA	23(1.16)	< 0.001
	IMV, *n* (%)	18(0.47)	33(0.91)	24(0.63)	23(0.59)	15(0.39)	18(0.52)	18(0.55)	15(0.44)	16(0.54)	15(0.75)	0.177
	NIMV, *n* (%)	56(1.47)	91(2.51)	73(1.91)	111(2.85)	102(2.68)	74(2.15)	52(1.58)	52(1.53)	72(2.43)	53(2.66)	< 0.001
	LOHS, mean (SD)	3.65(4.34)	3.7(4.02)	3.66(4.02)	3.8(4.61)	3.72(4.25)	3.89(4.75)	3.85(4.17)	3.84(5.16)	3.96(5.02)	4.04(5.29)	0.009
	IHM, *n* (%)	2(0.05)	2(0.06)	3(0.08)	2(0.05)	3(0.08)	1(0.03)	2(0.06)	5(0.15)	1(0.03)	1(0.05)	0.812
Both sexes	Pneumonia, *n* (%)	933(9.44)	715(7.45)	764(7.69)	811(8.28)	763(7.77)	661(7.85)	608(7.56)	613(7.53)	631(8.77)	302(6.25)	< 0.001
	Influenza, *n* (%)	75(0.76)	54(0.56)	42(0.42)	71(0.72)	63(0.64)	114(1.35)	40(0.5)	95(1.17)	104(1.45)	111(2.3)	< 0.001
	COVID-19, *n* (%)	NA	NA	NA	NA	NA	NA	NA	NA	NA	55(1.14)	< 0.001
	IMV, *n* (%)	63(0.64)	70(0.73)	57(0.57)	50(0.51)	55(0.56)	45(0.53)	42(0.52)	36(0.44)	45(0.63)	33(0.68)	0.371
	NIMV, *n* (%)	135(1.37)	206(2.15)	199(2)	243(2.48)	253(2.58)	178(2.11)	122(1.52)	135(1.66)	158(2.2)	116(2.4)	< 0.001
	LOHS, mean (SD)	3.55(4)	3.54(4)	3.46(3.66)	3.6(4.36)	3.52(3.99)	3.65(4.02)	3.67(3.77)	3.61(4.37)	3.73(5.17)	3.63(4.27)	0.062
	IHM, *n* (%)	5(0.05)	5(0.05)	7(0.07)	4(0.04)	11(0.11)	4(0.05)	4(0.05)	7(0.09)	2(0.03)	3(0.06)	0.546

*p* value for the trend from 2011 to 2020 (Cochran-Armitage tests for trend for categorical variables and Jonckheere-Terpstra test for LOHS)

*NA* not applicable, *IMV* invasive mechanical ventilation, *NIMV* non-invasive mechanical ventilation, *LOHS* length of hospital stay, *IHM* in-hospital mortality

Influenza was coded more frequently in recent years (1.9% of boys and 2.86% of girls in 2020 vs. 0.79% and 0.71%, respectively, in 2011; *p* < 0.001). In the year 2020, 55 children had codes for asthma and COVID-19 in their discharge report.

NIV was coded almost twice as frequently in 2020 (2.22% in boys and 2.66% in girls) as in 2011 (1.3% in boys and 1.47 in girls), with the trends being statistically significant for both sexes. IMV was used in < 1% and did not change over time.

The mean LOHS was slightly higher when an asthma diagnosis was recorded in any position (approx. 3.5 days) than when asthma was limited to the primary position (approx. 3.2 days). No significant change in the LOHS over time was found for either sex. Between 2011 and 2020, a total of 52 children (< 0.1%) died in hospital in Spain with asthma in any diagnostic position.

### Variables associated with IHM among children hospitalized with asthma in any diagnostic position

The results of the multivariable logistic regression model to identify which study variables were independently associated with IHM are shown in Table 4. Compared with the reference age group (0–5 years), children aged 6–10 years and 11–15 years were, respectively, 6.56 (95%CI 2.2–19.61) and 9.34 (95%CI 3.18–27.41) times more likely to die in hospital.

**Table 4:** Logistic regression model to identify factors associated with in hospital mortality among children hospitalized in Spain with asthma in any diagnosis position

**Variable**	**Categories**	**OR**	**95%CI**
Sex	Boy	1	-
	Girl	1.08	0.62–1.50
Age group	0–5 years	1	-
	6–10 years	6.56	2.2–19.61
	11–15 years	9.34	3.18–27.41
Pneumonia	No	1	-
	Yes	1.49	0.64–3.44
Influenza	No	1	-
	Yes	2.18	0.48–9.83
IMV	No	1	-
	Yes	36.08	20.72–62.84
NIMV	No	1	-
	Yes	0.56	0.13–2.43
Asthma as primary diagnosis	No	1	-
	Yes	0.37	0.17–0.7
Year of admission	Continuous	0.99	0.85–1.14

*IMV* invasive mechanical ventilation, *NIMV* non-invasive mechanical ventilation

Children who required IMV during their hospitalization also had a much higher risk of dying (OR 36.08; 95%CI 20.72–62.84).

Finally, when asthma was coded in the primary diagnostic position, the probability of dying during hospitalization was significantly lower (OR 0.37 95%CI 0.17–0.73).

## Discussion

Using the SNHDD, we found a significant decrease in rates of hospitalization of pediatric patients for asthma from 2011 to 2020. This decrease remained unchanged irrespective of whether asthma was analyzed only as primary diagnosis (asthma exacerbations) or in any diagnostic position. In addition, the frequency of hospitalizations was higher in boys than in girls for all the ages studied. NIV is increasingly common. IHM rates remained very low and stable over time. COVID-19 did not seem to increase admissions due to asthma in the year 2020.

The significant decrease in hospitalizations for asthma continued the trend already reported in a previous study, which included the entire Spanish population between the years 2002 and 2010 [11]. Similar results were found in studies conducted in other countries [23–28]. In Ecuador, Cabrera et al. observed that the hospitalization rates for asthma among children decreased from 27.8 to 12.9 per 100,000 inhabitants between 2000 and 2018 [23]. Similar works in high-income European countries also show reductions in hospitalization rates over the past three decades [24]. In other areas of the world, we see that decreases in hospital admission rates for asthma were of a different magnitude, although always consistent with the trend and period studied (Kuwait, 16%; Costa Rica, 53%; Brazil, 36%) [25–27].

In a recent study, admission rates were 3.38 times higher when admissions with any asthma diagnosis were used to calculate rates than with asthma as the primary diagnosis. These results highlight the importance of understanding the context of hospital administrative data collection in various settings, as there were substantial differences in hospital admission rates for asthma depending on the definition used, that is, whether asthma was recorded as a primary diagnosis or a secondary diagnosis [28]. In our study, we found that when the asthma code appeared in the first position, it was associated with a lower IHM. These results suggest that when asthma is not the first diagnosis, mortality was possibly due to the presence of other, more serious concomitant processes.

Consistent with other studies in other regions, our results indicate that COVID-19 did not cause an increase in admissions due to asthma [29, 30].

Mortality secondary to asthma in children is rare, ranging between 0.0 and 0.7/100,000 in different parts of the world, with Spain being among the lowest in Europe [15, 31–34]. In our study, we observed very low in-hospital mortality which remained largely unchanged from 2011 to 2020, although it did increase with age. Overrepresentation of adolescent males in pediatric asthma death series is consistent with data reported elsewhere [35]. Factors such as poorer adherence to medication and risk behaviors could explain, at least in part, the increase in mortality with age found in our study [36]. However, as we stated above, deaths recorded among older children may be related to other comorbid conditions or accidents, rather than to asthma.

Consistent with data reported elsewhere, we found that IMV was very infrequent and that NIV has replaced it as the primary mode of mechanical support for asthma [37].

It is important to highlight both the strengths and the limitations of our work. Its main strength is the large sample size and the use of a standardized methodology throughout the study. Similar studies have also used discharge databases to assess trends in hospital admissions for exacerbations in children with asthma [11, 38]. The main limitation of our study is the potential bias resulting from the use of ICD-9 and ICD-10 diagnostic codes to identify patients hospitalized for asthma. Previous studies found that “wheeze” or “bronchospasm” are commonly used instead of asthma in discharge reports, especially in younger children, and this could underestimate the real incidence of asthma [39]. In any case, other studies have used the same codes as we did to evaluate asthma hospitalization trends and have even performed sensitivity analyzes to address this issue, stating that it is difficult to conclude that the observed trend can be explained by diagnostic substitution [40]. Second, our data source was the SNHDD, an administrative database that does not include information on factors such as duration, severity, degree of control, or pharmacological treatments. Another limitation may derive from the fact that the database is anonymous, thus making it impossible to determine whether more than one admission corresponds to the same patient throughout the year. For the same reason, patients who moved from one hospital to another could appear more than once. Our findings show that the incidence of hospital admissions for asthma in Spain decreased in children between 2011 and 2020. We also recorded more frequent use of NIV and low mortality rates. This decrease is observed irrespective of whether asthma is analyzed as the primary diagnosis or in any diagnostic position. In addition, the frequency of hospitalizations is higher in boys than in girls for all the years studied, and the mean LOHS remained stable in both sexes. COVID-19 did not cause an increase in admissions with asthma in the year 2020. These findings indicate that management of patients with asthma improved in Spain during the study period.

## Supplementary Information

Below is the link to the electronic supplementary material.

Supplementary file1 (DOCX 14 KB)

## Data Availability

According to the contract signed with the Spanish Ministry of Health and Social Services, which provided access to the databases from the Spanish National Hospital Discharge Database, we cannot share the databases with any other investigator, and we must destroy the databases once the investigation has concluded. Consequently, we cannot upload the databases to any public repository. However, any investigator can apply for access to the databases by filling out the questionnaire available at http://www.msssi.gob.es/estadEstudios/estadisticas/estadisticas/estMinisterio/SolicitudCMBDdocs/Formulario_Peticion_Datos_CMBD.pdf. All other relevant data are included in the paper.

## Abbreviations

95%CI: 95% Confidence intervals
APC: Annual percentage change
ICD-10: International Classification of Diseases 10th edition
IHM: In-hospital mortality
IMV: Invasive mechanical ventilation
LOHS: Length of hospital stay
NIV: Non-invasive ventilation
OR: Odds ratios
SNHDD: Spanish National Hospital Discharge Database
