# Supplementary material for: Trends in hospital admissions among children with asthma in Spain (2011–2020)
Source: Eur J Pediatr. 2023 Mar 14;182(5):2409–19. doi: 10.1007/s00431-023-04873-w (PMC10011755; doi:10.1007/s00431-023-04873-w)
Supplement: Supplementary file 1 — Supplementary file1 (DOCX 14 KB) [file 431_2023_4873_MOESM1_ESM.docx]

Supplementary Table 1. Codes of the International Classification of Diseases 9^th^ and 10^th^ revisions used for this investigation

| **Diagnosis and procedures** | **ICID 9 Codes** | **ICD10 Codes** |
| --- | --- | --- |
| Asthma | 493 | J45 |
| Pneumonia | 480 to 486 | J12 to J18 |
| Influenza virus | 487 and 488 | J9 to J11 |
| COVID 19 | - | B34.2, B97.29, U07.1 |
| Invasive mechanical ventilation | 96.7, 96.70, 96.71, 96.72 | 5A1935Z 5A1945Z, 5A1955Z, |
| Non-invasive mechanical ventilation | 93.90 | 5A09357, 5A09457, 5A09557 |
